# Tinostamustine (EDO-S101) and Its Combination with Celecoxib or Temozolomide as a Therapeutic Option for Adult-Type Diffuse Gliomas

**DOI:** 10.3390/ijms26020661

**Published:** 2025-01-14

**Authors:** Wiktoria Pawlak, Aleksandra Majchrzak-Celińska

**Affiliations:** Department of Pharmaceutical Biochemistry, Poznan University of Medical Sciences, Rokietnicka 3, 60-806 Poznań, Poland; wiktoria.pawlak199@gmail.com

**Keywords:** glioma, astrocytoma, glioblastoma, tinostamustine, celecoxib, temozolomide, combinatory treatment

## Abstract

Adult-type diffuse gliomas are characterized by inevitable recurrence and very poor prognosis. Novel treatment options, including multimodal drugs or effective drug combinations, are therefore eagerly awaited. Tinostamustine is an alkylating and histone deacetylase inhibiting molecule with great potential in cancer treatment. Thus, the aim of this study was to investigate its effects on glioma cells. In this context, tinostamustine was evaluated in monotherapy and as a combination therapy, with either celecoxib or temozolomide; additionally, the results were compared to the golden glioma chemotherapy standard—temozolomide. Our experiments, conducted on both temozolomide-sensitive U-87 MG astrocytoma and temozolomide-resistant U-138 MG glioblastoma cells revealed that tinostamustine and its combination with either celecoxib or temozolomide exert dose-dependent cytotoxicity, cause cell cycle arrest, induce oxidative stress-mediated apoptosis of malignant glioma cells, and mitigate their migratory potential. Astrocytoma cells were more susceptible to the tested treatments than glioblastoma cells, and, generally, those dual therapies were superior in anti-glioma efficacy compared to temozolomide. Overall, our study provides evidence that tinostamustine and the combination therapies consisting of tinostamustine and celecoxib or tinostamustine and temozolomide may represent a new approach for the effective treatment of malignant gliomas.

## 1. Introduction

Gliomas are the most prevalent primary brain tumors in the central nervous system, with an average annual incidence of 4.5 per 100,000 [1]. They are a heterogeneous group of tumors that arise from glial cells in the brain and spinal cord. According to the 2021 WHO Classification of Tumors of the Central Nervous System, adult-type diffuse gliomas include astrocytoma *IDH*-mutant, oligodendroglioma *IDH*-mutant and 1p/19q-codeleted, and glioblastoma *IDH*-wildtype [2]. Clinically, the occurrence of *IDH1/2* mutations predicts longer survival and greater sensitivity to chemotherapy; thus, *IDH*-mutant astrocytomas carry significantly better prognosis as compared to glioblastomas [3]. WHO grade 4 *IDH*-mutant astrocytomas are also more radiosensitive than *IDH*-wildtype glioblastomas [4]. Another important biomarker for both astrocytoma and glioblastoma patients is O^6^-methylguanine DNA methyltransferase (*MGMT*) promoter methylation [5]. According to the literature review, patients with the methylated *MGMT* promoter live approximately 50% to 90% longer than those with an unmethylated *MGMT* promoter [6]. Thus, combined with *IDH1/2* mutations, *MGMT* promoter methylation status serves as an important prognostic marker for gliomas treated with radiation and chemotherapy [7]. Other clinically relevant glioma biomarkers are *TERT* promoter mutations and *PTEN* deletion, which indicate poor prognosis [8]. DNA methylation analysis also reveals aberrantly methylated genes which can be helpful in glioma patient stratification [9].

Despite differences in the molecular background of those different types of gliomas, their common features are a generally poor prognosis and almost inevitable recurrence [2]. As far as chemotherapy of gliomas is concerned, temozolomide (TMZ) has been the gold standard of the treatment for the last 20 years [10]. This drug penetrates the blood–brain barrier (BBB) and at physiologic pH is converted to its active metabolite, 5-(3-methyltriazen-1-yl) imidazole-4-carboxamide (MTIC), responsible for DNA alkylation and cancer cell death [11]. However, nearly half of glioblastoma patients treated with TMZ do not respond to the drug or develop treatment resistance during the course of treatment [12]. The overexpression of MGMT, as well as other resistance mechanisms, are responsible for the treatment failure and eventual tumor recurrence [13,14,15]. Among those, abnormal signaling pathways, autophagy, epigenetic changes, microRNAs, and the formation of extracellular vesicles have been identified as contributors to treatment resistance [13]. Taking all those challenges into consideration, novel treatment options for malignant gliomas are eagerly awaited.

Recently, there has been an intense interest in discovering multi-targeted antitumor drugs. One of those is tinostamustine (TINO or EDO-S101), an alkylating and histone deacetylase (HDAC) inhibiting molecule. It is a bifunctional compound that combines bendamustine with vorinostat in a new chemical entity. Its design was aimed to enhance the effectiveness of the alkylating DNA-damaging impact by inducing chromatin relaxation mediated by histone deacetylase. Thus, the presumed action of TINO involves alkylation of DNA, causing DNA crosslinking and double strand breaks, and, additionally, chromatin decondensation, leading to the activation of transcription of cell cycle inhibitors, both of which are expected to induce cell cycle arrest [16,17]. Its synthesis and pharmacological testing were first described in 2016, and since then, there has been a growing interest in exploring its anticancer potential in numerous cancer types [18].

Additionally, COXIBs, especially celecoxib (CELE), have also recently gained much attention for their chemotherapeutic potential in various cancer types, including central nervous system malignancies [19,20]. Our previous study demonstrated that in glioblastoma, CELE exerts its anticancer effect via Wnt signaling attenuation and COX-2/PGE2/EP4 pathway inhibition [19]. We also showed that CELE can ameliorate TMZ treatment [19]. Further studies also confirmed that CELE, in combination with TMZ, could mitigate the invasive characteristics of glioblastomas [21]. Interestingly, evidence also exists that the antitumor effect of HDAC inhibition can significantly be improved by the simultaneous inhibition of COX-2 [22]. Recently, even dual HDAC/COX-2 inhibitors are being designed and tested [23,24].

Thus, in this study, we hypothesized that TINO, in combination with CELE, might have a stronger anticancer effect as compared to TINO alone. Both TINO and CELE have been previously studied for therapeutic efficacy in glioblastomas, but combinations of these compounds have never been analyzed before. In this study, we also evaluated the combination of both TINO and TMZ, as well as compared their effects to TMZ alone. The effects of the analyzed compounds were assessed in terms of cytotoxicity, distribution of the cell cycle phases, apoptosis induction, reactive oxygen species (ROS) generation, and cell migration. For this purpose, we used two malignant glioma cell lines, U-87 MG astrocytoma and U-138 MG glioblastoma. Both of these cell lines are grade 4 gliomas; however, the U-87 MG cell line has an *MGMT* methylated promoter and is defined as TMZ sensitive, whereas the U-138 MG cell line has an *MGMT* unmethylated promoter and is regarded as TMZ resistant.

## 2. Results

### 2.1. TINO and Its Combination with CELE or TMZ Influence Glioma Cell Viability in a Concentration-Dependent Manner

**Figure 1:**
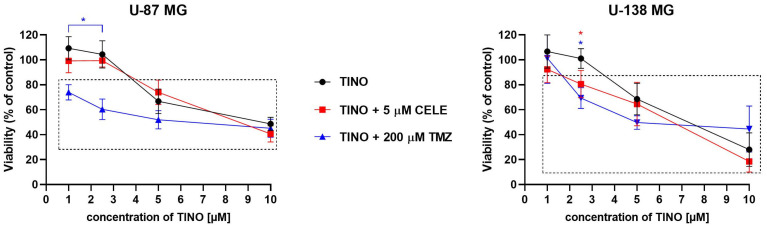
The viability (as expressed by the metabolic activity of cells) of U-87 MG and U-138 MG cell lines after 48 h of treatment with the analyzed compound/compounds. Cells were treated with the indicated drug concentrations of TINO or TINO and 5 µM CELE or 200 µM TMZ, and the viability was estimated using the MTT test. The square indicates statistically significant results as compared to the DMSO-treated control, whereas stars indicate statistically significant results for the drug combination (red for TINO + CELE and blue for TINO + TMZ) in respect to single treatment with TINO. Data are presented as mean values ± SD from three independent experiments.

The results of the MTT assay revealed that 48 h treatment with TINO decreases the number of living glioma cells in a concentration-dependent manner (Figure 1). In this regard, the lowest tested concentrations, i.e., 1 µM and 2.5 µM, were not cytotoxic, but 5 µM TINO significantly decreased the number of living cells of both U-87 MG and U-138 MG cell lines, leaving ~70% of metabolically active cells. However, the highest tested concentration, i.e., 10 µM reduced the number of living cells to 48.6 ± 5.4% and 28.0 ± 13.5% of U-87 MG and U-138 MG cells, respectively. Next, we wanted to analyze if the addition of a fixed, non-toxic concentration of CELE (5 µM) or a fixed concentration of TMZ of 200 µM (U-87 MG cells are sensitive, whereas U-138 MG cells are resistant to TMZ in this concentration) enhances the cytotoxicity of TINO treatment. In this context, the most spectacular results were observed for the combination of 1 µM TINO and 200 µM TMZ in the U-87 MG cell line and 2.5 µM TINO and 200 µM TMZ for both U-87 MG and U-138 MG cell lines. Those combinations were more cytotoxic as compared to the DMSO-treated control and as compared to single TINO treatment with the respective concentration. Moreover, in U-138 MG cells, the combination of 2.5 µM TINO and 5 µM CELE was also significantly more cytotoxic as compared to both the DMSO-treated control and single treatment with 2.5 µM TINO.

Based on the MTT results the concentration of 5 µM of TINO (as the closest to the IC25, allowing the survival of ~75% of cells) was chosen for further analyses.

### 2.2. The Combinations of TINO and CELE as Well as TINO and TMZ Halt the Cell Cycle of Glioma Cell Lines

**Figure 2:**
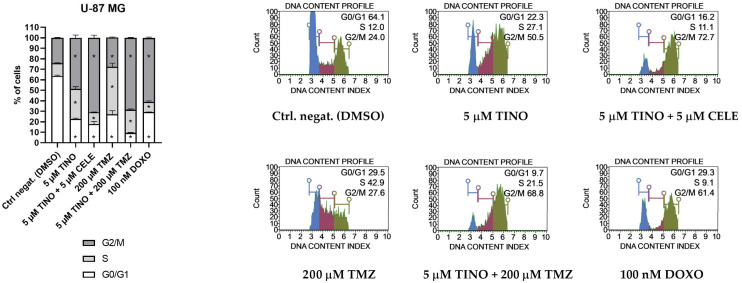
Cell cycle analysis of U-87 MG astrocytoma cell line using the Muse Cell Cycle Kit (Merck, Darmstadt, Germany) after 48 h of treatment. DMSO and doxorubicin (DOXO) were used as negative and positive controls of this assay, respectively. The percentages of cells in the G0/G1, S, and G2/M phases were analyzed by flow cytometry after staining with propidium iodide and RNase A. Values are expressed as mean ± SD from at least two independent experiments. Asterisks indicates the values that are significantly different from the DMSO-treated control (*p* < 0.05 was considered statistically significant). Representative histograms (with G0/G1, S and G2 phases indicated with blue, purple, and green color, respectively) are also presented.

**Figure 3:**
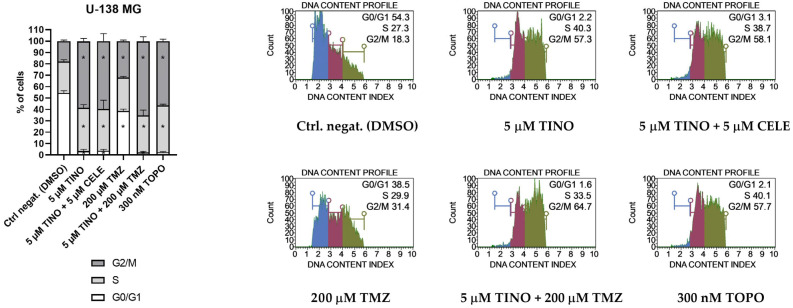
Cell cycle analysis of U-138 MG glioblastoma cell line using the Muse Cell Cycle Kitafter 48 h of treatment. DMSO and topotecan (TOPO) were used as negative and positive controls of this assay, respectively. The percentages of cells in the G0/G1, S, and G2/M phases were analyzed by flow cytometry after staining with propidium iodide and RNase A. Values are expressed as mean ± SD from at least two independent experiments. Asterisks indicates the values that are significantly different from the DMSO-treated control (*p* < 0.05 was considered statistically significant). Representative histograms (with G0/G1, S and G2 phases indicated with blue, purple, and green color, respectively) are also presented.

Next, we wanted to verify if the distribution of the cell cycle phases of glioma cell lines is altered by the 48 h treatment with 5 µM TINO and its combination with fixed non-toxic concentrations of CELE (5 µM) or TMZ (200 µM) (Figure 2 and Figure 3). Single treatment with 200 µM TMZ was also tested for reference. As far as the U-87 MG astrocytoma is concerned, the most pronounced changes were observed after the treatment with the 5 µM TINO + 5 µM CELE and 5 µM TINO + 200 µM TMZ combinations. These combinations halted the cell cycle in the G2/M restriction point, and the distribution of cell cycle phases resembled what was obtained after 100 nM doxorubicin (DOXO) treatment. Cell cycle arrest was also observed after the treatment with only 5 µM TINO. The treatment with TMZ in a concentration of 200 µM (the IC50 for this cell line is 223.1 μM [25]) was accompanied by a decrease in the percentage of cells in the G0/G1 phases, an increase in the percentage of cells in the S phase, and also a slight (but significant) increase in the number of cells in the G2/M phases.

Regarding the U-138 MG glioblastoma cell line, our data revealed that 5 µM TINO and its combinations with 5 µM CELE or 200 µM TMZ are very efficient in halting the cell cycle, similar to the action of DOXO. The distribution of the cell cycle phases of samples exposed to TINO and its combinations with CELE or TMZ were characterized by a huge decrease in the number of cells in the G0/G1 phases, followed by a modest increase in the number of cells in the S phase and a large increase in the population of cells in the G2/M phases. On the other hand, the treatment with 200 µM TMZ resulted in a significant decrease in the number of cells in the G0/G1 phases and an increase in the number of cells in the G2/M phases, while the population of cells undergoing DNA replication did not differ as compared to the DMSO-treated control.

### 2.3. The Treatment with TINO or Its Combination with CELE or TMZ Induces Massive Oxidative Stress in Glioma Cells

**Figure 4:**
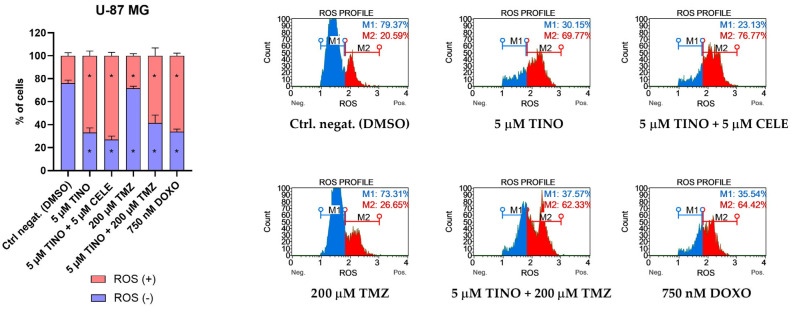
Oxidative stress analysis of U-87 MG astrocytoma cell line using the Muse Oxidative Stress Kit (Merck, Darmstadt, Germany) after 48 h of treatment. DMSO and doxorubicin (DOXO) were used as negative and positive controls of this assay, respectively. ROS (+) indicates a cell population with detectable superoxide radicals, whereas ROS (-) indicates cells without detectable superoxide radicals. Values are expressed as mean ± SD from at least two independent experiments. Asterisks indicates the values that are significantly different from the DMSO-treated control (*p* < 0.05 was considered statistically significant). Representative histograms are also presented.

Subsequently, using the flow cytometry method, we analyzed the intracellular level of superoxide radicals in cells undergoing the treatment with the analyzed compound/s. Regarding the U-87 MG astrocytoma cell line, we observed a significant increase in the cellular population undergoing oxidative stress following the treatment with both TINO and its combinations (Figure 4). In each case when TINO was evaluated (either as a single treatment or in a combination), the number of cells with a detectable level of superoxide radicals exceeded 60%. The treatment with 200 µM TMZ only slightly increased the percentage of cells undergoing oxidative stress.

**Figure 5:**
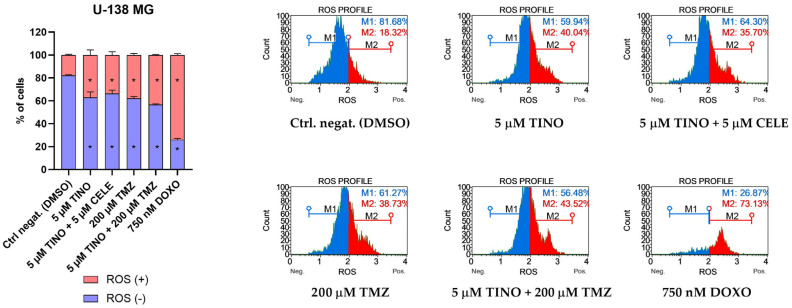
Oxidative stress analysis of U-138 MG glioblastoma cell line using the Muse Oxidative Stress Kit after 48 h of treatment. DMSO and doxorubicin (DOXO) were used as negative and positive controls of this assay, respectively. ROS (+) indicates a cell population with detectable superoxide radicals, whereas ROS (-) indicates cells without detectable superoxide radicals. Values are expressed as mean ± SD from at least two independent experiments. Asterisks indicates the values that are significantly different from the DMSO-treated control (*p* < 0.05 was considered statistically significant). Representative histograms are also presented.

Our results revealed that in U-138 MG glioblastoma cells, the tested compounds induced oxidative stress; although, the results were not so pronounced as compared to the U-87 MG cells (Figure 5). Here, we observed very similar results when TINO was used alone or in a combination—~40% of cells underwent oxidative stress. Importantly, the addition of TINO to TMZ created a higher level of intracellular superoxide radicals as compared to TMZ administered alone.

### 2.4. The Pro-Apoptotic Effects of TINO and Its Combinations Are More Pronounced in Astrocytoma as Compared to Glioblastoma Cells

**Figure 6:**
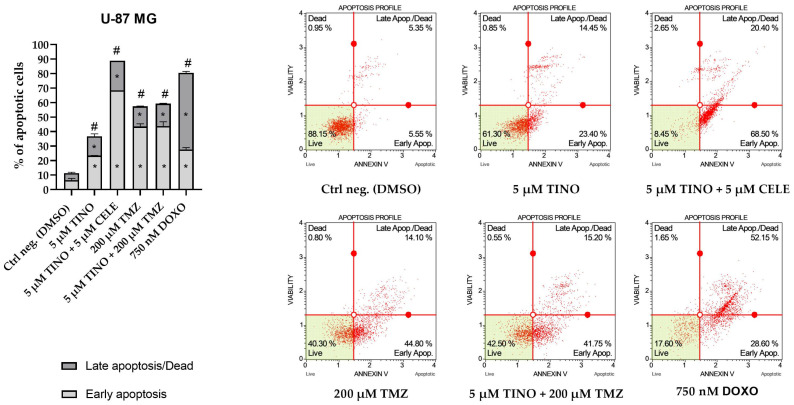
The results of apoptosis analysis obtained using Muse ™ Annexin V & Dead Cell Kit (Merck, Darmstadt, Germany) on U-87 MG astrocytoma cell line. DMSO and doxorubicin (DOXO) were used as negative and positive controls of the assay, respectively. The bar chart represents the percentage of apoptotic cells after 48 h of treatment with the compound/compounds. The values are shown as the mean ± SD calculated from at least two independent experiments. The star (*) indicates a statistically significant difference as compared to the DMSO-treated control for early/late apoptosis. A hashtag (#) above the bar indicates statistically significant differences as compared to the DMSO-treated control for total apoptotic cells (*p* < 0.05 was considered statistically significant). Representative histograms are also presented.

Next, we analyzed if the treatment with TINO or its combination with CELE or TMZ induce apoptotic cell death—the final goal of any anticancer treatment. The results showed that after 48 h incubation, all the treatments successfully induced apoptosis of U-87 MG astrocytoma cells; the strongest pro-apoptotic effect was, however, observed after the treatment with the combination of 5 µM TINO and 5 µM CELE—almost 90% of cells were either early or late apoptotic (Figure 6). The second most pronounced results were obtained after the treatment with 5 µM TINO and 200 µM TMZ or a single treatment with 200 µM TMZ. Both treatments resulted in the induction of apoptosis in more than half of the exposed cells. Treatment with only 5 µM TINO also resulted in a statistically significant pro-apoptotic effect, with ~4 times more apoptotic cells as compared to the negative control (DMSO-treated cells).

**Figure 7:**
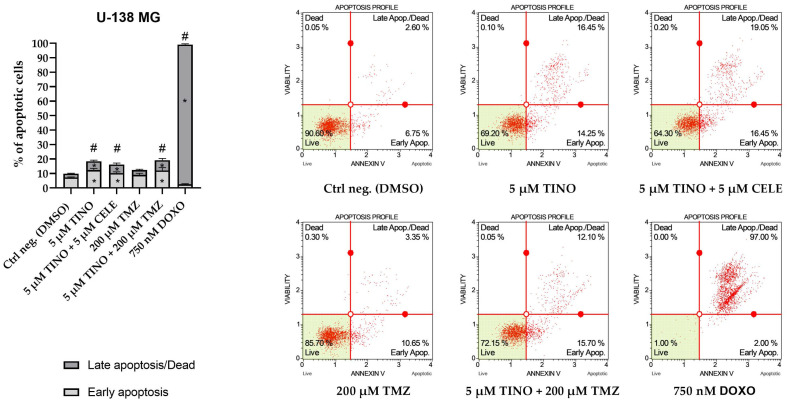
The results of apoptosis analysis obtained using Muse ™ Annexin V & Dead Cell Kit on U-138 MG glioblastoma cell line. DMSO and doxorubicin (DOXO) were used as negative and positive controls of the assay, respectively. The bar chart represents the percentage of apoptotic cells after 48 h of treatment with the compound/compounds. The values are shown as the mean ± SD calculated from at least two independent experiments. The star (*) indicates a statistically significant difference as compared to the DMSO-treated control for early/late apoptosis. A hashtag (#) above the bar indicates statistically significant differences as compared to the DMSO-treated control for total apoptotic cells (*p* < 0.05 was considered statistically significant). Representative histograms are also presented.

As far as U-138 MG glioblastoma cells are concerned, the results obtained after the treatment with TINO and its combinations were also statistically significant, as compared to the negative control of the assay; however, they were less pronounced in comparison with the effects observed in astrocytoma cells (Figure 7). Generally, the treatment with 5 µM TINO and its combinations doubled the number of apoptotic cells as compared to the DMSO-treated control. In this cell line, 200 µM TMZ did not induce a statistically significant pro-apoptotic effect. On the other hand, the most spectacular pro-apoptotic effects were observed for our positive control of the assay, DOXO, which induced apoptosis in practically all of the treated cells.

### 2.5. Combination of TINO with TMZ and TINO with CELE Significantly Limits the Migratory Potential of Glioblastoma Cells

**Figure 8:**
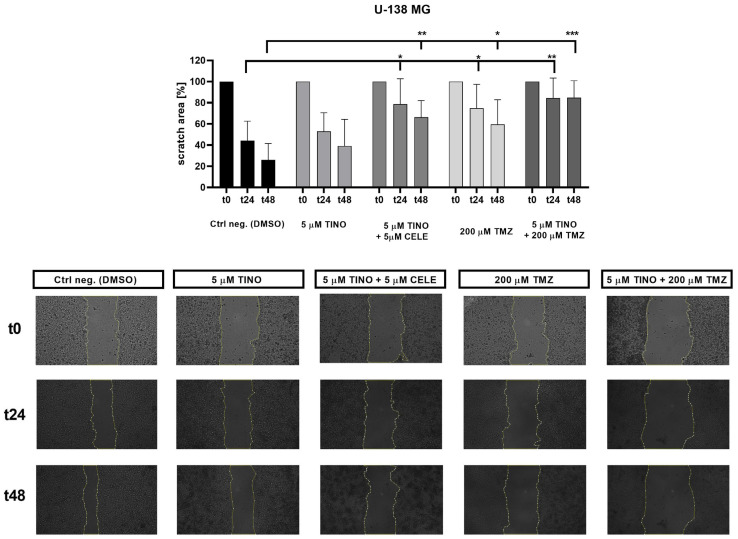
Scratch assay results of the U-138 MG glioblastoma cell line. The scratch area was measured at the initial t0 and following 24 and 48 h of incubation with the analyzed compound/s (indicated as t24 and t48, respectively). The results from each time point were statistically evaluated in respect to the data obtained from the DMSO-treated control. A single star (*) denotes the level of statistical significance with a *p* value of 0.01–0.05; two stars (**) denotes *p* = 0.001–0.01, whereas three stars (***) denotes *p* = 0.0001–0.001. The graph presents the pooled data from at least two independent experiments with four measurements per assay. The pictures below present the results from one single experiment.

Adult diffuse gliomas are characterized by high migratory potential [26]. This growth pattern is one of the major factors contributing to therapeutic failure. Therefore, we wanted to check to what extent TINO and its combination with CELE and TMZ influence glioblastoma cell migration. The scratch assay performed using U-138 MG cells revealed that TINO combinations limit the migration capacity more potently than TINO alone (Figure 8). The highest anti-migratory potential was obtained when the combination of 5 µM TINO and 200 µM TMZ was administered. The difference in the scratch area of this sample as compared to the control reached statistical significance with *p* = 0.0092 and *p* = 0.0004 for t24 and t48, respectively. In this case, the scratch area decreased only ~15% during the first 24 h and did not shrink during the next 24 h. The migratory ability of cells was also diminished following the treatment with 5 µM TINO and 5 µM CELE and resembled the effect obtained after the treatment with 200 µM TMZ.

## 3. Discussion

In this manuscript, we provide evidence that TINO and its combination with other drugs, such as CELE or TMZ, should be further evaluated as a promising new treatment strategy for adult-type diffuse gliomas.

In our study, we evaluated the impact of TINO in a concentration range of 1–10 µM on two glioma (one astrocytoma and one glioblastoma) cell lines, showing concentration-dependent cytotoxicity. The highest tested concentration was 10 µM, and in both cell lines, it exerted significant toxicity. This can be regarded as a comparable or even better result as opposed to the TMZ active concentration reached in the cerebrospinal fluid of glioma patients, following the standard regimen of 150–200 mg/m^2^/day. In this regard, Patel et al. found that the administration of TMZ in this dose range results in peak concentrations of 3 to 15 μg/mL (15–77 μM) in the plasma of non-human primates [27,28], while further studies performed by Ostermann et al. revealed that only one fifth (20 ± 5%) of the TMZ plasma concentration reaches the cerebrospinal fluid [28,29]. Considering that TINO has an excellent central nervous system penetration of 13.8% and 16.5% by intravenous infusion and bolus administration, respectively [30], we can assume it is way more potent as compared to TMZ.

Nevertheless, since the vast majority of in vitro and in vivo studies on TMZ elucidate much higher concentration ranges of TMZ (hundreds µM), we decided to use the concentration of 200 µM as the “golden mean” concentration for the U-87 MG, TMZ sensitive and U-138 MG, TMZ resistant cell lines. Thus, comparing those pharmacologically active concentrations of TINO and TMZ in our study, we observed comparable or even better anti-glioma effects of TINO, which was striking, knowing that TINO was used in a concentration 40 times lower than TMZ. We assume that this spectacular difference results from the fact that in the case of TINO, there are two pharmacophores in one molecular structure, which makes it possible to reduce the pharmacologically active dose (and hypothetically also the side effects for the patients). This is an important finding, especially for those patients who initially are or become resistant to TMZ therapy during the course of treatment. Additionally, Festuccia et al. provided interesting evidence that TINO is synergistic with radiotherapy [31]. They demonstrated that TINO possesses stronger antiproliferative and pro-apoptotic effects than those observed for vorinostat and bendamustine alone, and the results are similar for their combination irrespective of MGMT expression [31]. Importantly, TINO prolonged the disease-free survival and overall survival of mice, and the effect was superior as compared to bendamustine, radiotherapy, and TMZ [31].

Another important aspect covered in our study was the anti-glioma potential of combination therapy, consisting of TINO and CELE or TINO and TMZ. To the best of our knowledge, such combinations have never been evaluated before. Thus, for the first combination, we chose 5 µM TINO and 5 µM CELE (this concentration of CELE is regarded as non-toxic to both of the tested cell lines), and for the second combination, we evaluated 5 µM TINO and 200 µM TMZ.

The idea of combining TINO with CELE came out from recent data, suggesting potential additive or synergistic effects of HDACi and COX inhibitors. Such effects were observed in salivary adenoid cystic carcinoma as well as tongue squamous cell carcinoma, but it can be assumed that in other cancer types, including glioma, those effects might also be present [32,33]. Taking into consideration that HDACs and COX-2 are overexpressed in several cancer types, recently, libraries of dual inhibitors of these two enzymes are being created [24]. In a study by Liu et al., a novel bioactive hybrid of CELE with HDACi was found active in human acute lymphoblastic leukemia cells, inducing apoptosis by activating PARP cleavage [23]. Additionally, in a study by Zhang and Gan, the synergistic antitumor effects of the combined treatment with an HDAC6 inhibitor and a COX-2 inhibitor were exerted by activating the PTEN/AKT signaling pathway [33].

In our study, we performed the phenotypic screening of glioma cells following the treatment with HDACi-alkylating TINO and COX-2 inhibiting CELE. Our study revealed that this combination exerted stronger anti-glioma effects as compared to TINO used in monotherapy or TMZ used in monotherapy, especially in the U-87 MG cell line. In this context, TINO and CELE induced the strongest ROS-generating and pro-apoptotic effect in the U-87 MG cell line. This combination also induced pronounced cell cycle arrest in this cell line. However, as far as U-138 MG glioblastoma cells are concerned, the combination of TINO and CELE was very active, but the results were comparable to those obtained after TINO monotherapy. On the other hand, the scratch assay revealed that combining TINO and CELE result in stronger mitigation of cell migration as compared to TINO, which in this assay did not produce statistically significant changes. Thus, we can conclude, based on this phenotypic screen, that astrocytoma cells are generally more susceptible to this combination therapy, as compared to glioblastoma cells. However, the invasion of the latter cells might be successfully diminished by this combination. Further studies are, however, necessary to fully verify that these observations are valid in other glioma cell line models and also in animal experiments.

Next, we verified the effects of the combination of TINO and TMZ as a dual therapy against malignant glioma. In this regard, numerous studies reveal that dual therapies provide better effects than using TMZ alone. For instance, a synergistic effect of TMZ and DOXO has recently been found [34]. In a study of Dhungel et al., the above-mentioned combination enhanced DOXO uptake and induced higher apoptosis in TMZ-resistant GBM43 cells [34]. Also, the latest study by Oraiopoulou et al. suggests that a TMZ–DOXO dual chemotherapeutic scheme disables proliferation and increases cytotoxicity against glioblastoma cells [35]. Other combinations, i.e., consisting of TMZ and perifosine (allosteric AKT inhibitor) are evaluated, providing evidence that the two compounds synergistically inhibit glioblastoma by impeding DNA repair and inducing apoptosis [36]. In another study, the combination therapy of TMZ and CELE showed potent inhibitory effects on the TMZ-resistant GBM cell lines LN229 and LN18 [37]. The authors showed that this combination therapy inhibits cell proliferation and increases apoptosis and autophagy in LN229 and LN18 cell lines, and those effects were related to mitochondrial metabolism and respiratory chain inhibition [37]. Other drug combinations are also being explored, with promising results.

Those and other observations allowed us to hypothesize that dual therapy, consisting of TINO and TMZ, might also be more effective against glioma cells than TMZ alone. In this context, we found that such a treatment brings benefits as compared to TMZ monotherapy and similar effects as compared to TINO and CELE dual therapy. The most spectacular results of this combination were observed in the scratch assay, where the wound only modestly decreased during the first 24 h following the treatment with TINO and TMZ and remained practically unchanged during the next 24 h. It is important to note that, according to some evidence, particular existing anti-glioma therapies, including ionizing radiation and VEGF blockers, can sometimes increase GBM invasion [38,39,40,41]. In this context, the anti-migratory potential of TINO and TMZ dual therapy is of particular importance. Additionally, in our study, TINO and TMZ induced pronounced oxidative stress in U-87 MG cells, whereas TMZ only modestly increased the number of ROS (+) cells. Nevertheless, in this cell line, both TINO and TMZ, as well as TMZ monotherapy, induced similar effects in regard to apoptosis induction—the apoptotic process was detected in both cases in ~60% of cells. On the other hand, in U-138 MG glioblastoma cells, the combination therapy induced a pro-apoptotic effect, whereas TMZ monotherapy did not provide statistically significant results. Thus, we can conclude that the addition of TINO to TMZ therapy can be taken into consideration when TMZ is or becomes ineffective. The safety profile of such a combination remains, however, to be elucidated.

Interestingly, in a recent study by Pak et al., the combination of another HDACi, valproic acid, with CELE enhanced the effect of TMZ on glioblastoma cells. This drug combination was also very active in vivo, reducing the tumor size and prolonging the survival of experimental animals [42]. Thus, the next studies combining TINO, CELE, and TMZ are also worth elucidation, with a focus on their underlying biological mechanisms. In a study by López-Iglesias et al., the HDACi effect of TINO was demonstrated by α-tubulin and histone hyperacetylation, while a DNA-damaging effect was shown by an increase in γH2AX [43]. Moreover, using a reporter plasmid integrated into the genome of some multiple myeloma cell lines, they demonstrated that, apart from inducing potent DNA damage, TINO specifically inhibited double-strand break repair by the homologous recombination pathway. Moreover, TINO treatment reduced the recruitment of repair proteins such as RAD51 to DNA-damage sites identified as γH2AX foci. Such mechanistic studies are needed in regard to TINO and its combinations in glioma cells.

It is important to note the limitations of this study, which include working with only selected concentrations of drugs, limiting the possibility to clearly define the type of combinatorial effect (synergistic or additive) of those dual therapies. Additionally, in vivo models are needed to validate our findings in the physiological context and confirm the clinical utility of the above-mentioned drug combinations. Another aspect to be elucidated is whether TINO is a substrate for the efflux pumps present in the BBB and the tumor cells. The data regarding gliomas are scarce, but in a study by Chesi et al., TINO was the only drug to exhibit activity as a single agent in the multidrug-resistant transplant model of relapsed/refractory multiple myeloma [44]. The efficacy of TINO in the context of glioma patients expressing ABC transporters needs to be clarified. Evaluation of the long-term effects of such a therapy and the analysis of the risk of potential resistance development are other aspects that should be covered in future studies.

Currently, there is an ongoing open-label, multi-center, phase 1 study (ClinicalTrials.gov ID NCT05432375) of TINO, used as an adjuvant treatment in patients with newly diagnosed glioblastoma who are *MGMT* unmethylated and have completed concomitant treatment with TMZ and radiation. The study investigates the safety, pharmacokinetics, and efficacy of TINO. Hopefully, it will reveal the great potential of TINO in the clinical setting. Moreover, based on the data gathered in this study, we can conclude that not only TINO but also its combinations with CELE or TMZ are promising new therapeutic options for malignant glioma patients and should be further intensively evaluated.

## 4. Materials and Methods

### 4.1. Cell Lines and Culture

U-138 MG and U-87 MG were purchased from American Type Culture Collection (ATCC, Gaithersburg, MD, USA). Cells were maintained in a 37 °C humidified atmosphere containing 5% CO_2_ in Eagles Minimum Essential Media (EMEM) (Gibco, ThermoFisher Scientific, Waltham, MA, USA), supplemented with 10% fetal bovine serum (EURx, Gdansk, Poland) and antibiotics, i.e., penicillin and streptomycin (Merck, Darmstadt, Germany) to final concentrations of 1%.

U-87 MG cells express low level of MGMT due to partial methylation of the promoter and are sensitive to TMZ. On the other hand, U-138 MG cells express MGMT protein (they have an unmethylated *MGMT* promoter) and are TMZ resistant [12].

### 4.2. Compounds

TINO was obtained from MedChemExpress (1 Deer Park Dr, Suite Q, Monmouth Junction, NJ 08852, USA), while CELE and TMZ were purchased from Merck (Darmstadt, Germany). The compounds were dissolved in DMSO, aliquoted, and stored at −20 °C until needed.

### 4.3. Cell Viability Assay

The effect of TINO and its combination with CELE or TMZ on U-87 MG and U-138 MG cell lines’ viability was assessed using the MTT method. The assay was performed according to the standard procedure. Briefly, cells were seeded at a density of 10,000 cells per well in 96-well plates and cultured for 24 h to allow cell attachment. Afterwards, TINO or TINO and CELE or TINO and TMZ were added (TINO in a concentration range of 1–10 µM, whereas CELE and TMZ were always in a fixed concentration of 5 µM and 200 µM, respectively). DMSO-treated cells served as a control. After 48 h incubation, the cells were washed with phosphate-buffered saline (PBS) and incubated again for 4 h in a medium supplemented with 0.5 mg/mL MTT (3-[4,5-dimethylthiazole-2-yl]-2,5-diphenyltetrazolium bromide). Next, the medium containing the MTT solution was removed, and formazan crystals were dissolved in acidic isopropanol. Finally, the absorbance at λ = 570 nm and λ = 690 nm was measured on Tecan Infinite M200 microplate reader (Grödig, Austria). Cell viability was calculated as the relative percentage of the DMSO-treated control. All treatments were performed in triplicate with four measurements per assay. The concentration of 5 µM TINO was chosen for further analysis, as it was for both the single treatment and the combinations closest to the IC25 value.

### 4.4. Cell Cycle Analysis

For cell cycle analysis, the cells were seeded at a density of 50,000 cells/well in 12-well plates and incubated for 24 h. TINO or its combination with CELE or TMZ were added to cells and left for 48 h incubation. Afterwards, cells were harvested by trypsinization, fixed in ice-cold 70% ethanol, and stored at −20 °C until staining. After overnight storage, cells were stained with propidium iodide in the presence of RNase A and analyzed by flow cytometry on the Muse^®^ Cell Analyzer (Merck, Darmstadt, Germany). Utilizing the Muse^®^ 1.5 Analytical Software (Merck, Darmstadt, Germany), the experiment results were examined.

### 4.5. Apoptosis Analysis

A total of 50,000 cells per well were seeded in 12-well plates and incubated for 24 h to allow the cells to attach to the bottom of the plate. The tested compounds or their combination were added to the cells, and they were further incubated for 48 h. Cells were collected by trypsinization and stained with annexin V and 7-aminoactinomycin D (7-AAD) solution, being part of the Annexin V and Dead Cell kit (Merck, Darmstadt, Germany). The cells were then analyzed by flow cytometry on the Muse^®^ Cell Analyzer (Merck, Darmstadt, Germany). Utilizing the Muse^®^ 1.5 Analytical Software (Merck, Darmstadt, Germany), the experimental results were examined.

### 4.6. Scratch Assay

The scratch assay was performed according to the guidelines of Cormier et al., with some modifications [45]. A confluent (~95% of confluency) monolayer of cells was created by seeding 50,000 cells per well in 96-well plates. After 24 h of incubation, allowing the cells to attach to the bottom of the plate, a horizontal scratch was created in each well using a sterile 10 µL pipet tip. After creating the scratch, the medium was aspirated, and the cells were rinsed with prewarmed PBS. Fresh medium with DMSO or medium containing the analyzed compounds or their combinations were added to the wells, and the initial images of the scratch area (t0) were taken of each well using the Millicell^®^ DCI Digital Cell Imager (Merck, Darmstadt, Germany). After 24 and 48 h of incubation, the images were collected again (t24 and t48, respectively) and were analyzed using ImageJ software, version 1.8.0. The experiment was repeated at least twice with four wells per analyzed compound/s, per assay. The data from repeated wells were pooled, and the total amount of migration was calculated by subtracting the final migration area for each test group from the average premigration area of the control samples.

### 4.7. Statistical Analysis

Statistical analysis was carried out using the Student’s *t*-test (two-tailed). All data measurements were reported as the mean ± SD. *p* < 0.05 was considered statistically significant.

## 5. Conclusions

TINO, with its dual mechanism of action consisting of DNA alkylation and HDAC inhibition, is a highly interesting anticancer molecule per se, but combined with COX-2 inhibitor or with classical chemotherapy, it might offer even more effective treatment outcomes for adult-type diffuse gliomas. In this study, we provided evidence that both tested drug combinations, namely TINO and CELE as well as TINO and TMZ, were found to be superior in terms of the anticancer effects as compared to the currently used, gold standard, TMZ. This finding is especially important in respect to glioma patients who initially are, or become, TMZ resistant and lack effective treatment options. Additional studies using animal models and clinical trials are, however, needed to fully determine the safety and efficacy of those combinations.

## Data Availability

The results supporting the reported results can be obtained from the authors upon request.
